# The relationship between serum lipid levels and colorectal serrated lesions: A systematic review and meta-analysis

**DOI:** 10.3389/fphys.2022.984586

**Published:** 2022-10-11

**Authors:** Xuerui Wang, Yangbin Zou, Ruxuan Zhang, Chunyan Teng, Xuejiao Ren, Haishan Zhang, Liting Zhou

**Affiliations:** ^1^ Department of Gastrointestinal Colorectal and Anal Surgery, China-Japan Union Hospital of Jilin University, Changchun, China; ^2^ Department of Clinical Laboratory, The Second Hospital of Jilin University, Changchun, China; ^3^ Department of Occupational and Environmental Health, School of Public Health, Jilin University, Changchun, China

**Keywords:** colorectal polyp, sessile serrated adenoma, meta-analysis, lipid, colorectal serrated lesions

## Abstract

**Objective:** To clarify the relationship between colorectal serrated lesions and serum lipid levels, and provide a scientific basis for the identification and early clinical prevention and treatment of populations that are at risk for colorectal serrated lesions.

**Methods:** Studies comparing serum lipid levels in patients with colorectal serrated lesions and controls were searched in PubMed, Embase, Web of Science, the Cochrane Library, China Biomedical Literature Database, CNKI, Wanfang Database, and VIP Database. Relevant literature was screened according to the inclusion and exclusion criteria. The mean and standard deviation of the serum lipid levels in patients and controls were extracted from the included literature. The combined weighted mean difference (WMD) and 95% confidence intervals (CIs) were calculated using Review Manager 5.0 software to evaluate the relationship between serum lipid levels and colorectal serrated lesions. Publication bias of the included studies was evaluated by the Egger test.

**Results:** Twenty-three studies were included, comprising 2,063 patients and 63,909 controls. The serum high-density lipoprotein cholesterol (HDL-C) levels in the case group was significantly lower than in the control group (WMD = −0.122 mmol/L, 95% CI: 0.170–0.073). Total cholesterol (TC), low-density lipoprotein cholesterol (LDL-C), and serum triglyceride levels in the case group were significantly higher than in the control group, and the WMDs were 0.180 mmol/L (95% CI: 0.061–0.299), 0.155 mmol/L (95% CI: 0.038–0.273), and 0.241 mmol/L (95% CI: 0.181–0.302), respectively.

**Conclusion:** Colorectal serrated lesions may be related to blood lipid levels. Hyperlipidemia might be a risk factor for colorectal serrated lesions.

## Introduction

Colorectal cancer (CRC) is one of the most common malignant tumors in the world. At present, the incidence and mortality rates of CRC are the third and second of all cancers, respectively (Sung et al., 2021). According to the Global Cancer Statistics Report 2020, there were 1,931,590 new CRC cases and 935,173 deaths worldwide in 2020, accounting for 10.0% and 9.4% of cancer cases and deaths, respectively. In China, the incidence of CRC is second only to lung cancer, and the standardized death rate from CRC in both men and women in China is higher than the global average (WHO, 2021). CRC has become a major public health problem affecting people health.

Colorectal adenoma (CRA) is the most important precancerous lesion of CRC, and about 80% of CRC progresses from CRA (Zhao et al., 2021). CRA includes traditional adenomas and serrated lesions s, both of which may progress to CRC through different mechanisms. Traditional adenomas include tubular adenomas, villous adenomas, and tubular villous adenomas. They can progress to CRC through the traditional adenoma-carcinoma pathway, which is generally considered to be the main progress pathway for CRC (Dekker et al., 2019). Colorectal serrated lesions include hyperplastic polyps, sessile serrated adenomas/polyps (SSA/P), and traditional serrated adenomas (TSA). Among them, SSA/P and TSA can progress to CRC through serrated adenoma cancer (Hawkins et al., 2002), which is another morphological multistep pathway parallel to the traditional adenoma-cancer pathway. About one-third of CRC progresses through it (Pai et al., 2019). The pathogenesis of serrated colorectal lesions and polyposis mainly involves BRAF serrated pathway and KRAS serrated pathway. Patients with SSA/P or TSA have higher mutation rates of KRAS and BRAF, and tumor cells are more prone to excessive proliferation, therefore patients with SSA/P or TSA have a significantly higher risk of CRC than those with traditional adenomas (Erichsen et al., 2016). In addition, colorectal serrated lesions are more common in people over 50-years-old, and the detection rate is increasing yearly (Anderson and Srivastava, 2020). Therefore, distinguishing colorectal serrated lesions from traditional adenomas and identifying easily detectable risk factors of colorectal serrated lesions can help identify people at high risk for CRC.

Hyperlipidemia, obesity, and metabolic syndrome are considered risk factors for CRC (Coppola et al., 2015). Studies have shown that hyperlipidemia was an independent risk factor for adenomatous polyps (Macarie et al., 2020). Obesity, smoking, dietary fat, total energy intake, and red meat intake have been associated with an increased risk of colorectal serrated lesions (Wallace et al., 2009; Bailie et al., 2017). Obesity has been directly related to the occurrence of colorectal serrated lesions (Haque et al., 2014). Serum lipid level is a routine index of clinical physical examinations and is closely related to hyperlipidemia, obesity, and metabolic syndrome.

Lipid levels may be associated with the risk of colorectal serrated lesions, but the results of existing studies are inconsistent. Tabuchi et al. (Tabuchi et al., 2006) found that there were no significant differences in serum total cholesterol (TC) and triglyceride (TG) between patients with colorectal serrated lesions and those without serrated lesions. Pyo et al. (Pyo et al., 2018) found that the serum TG level was an independent risk factor for TSA. A study by Fliss-Isakov et al. suggested that serum high-density lipoprotein cholesterol (HDL-C) was significantly decreased in female patients with colorectal serrated lesions (Fliss-Isakov et al., 2017). Therefore, the relationship between colorectal serrated lesions and the level of blood lipids needs evidence-based analysis. The present study was a systematic review and meta-analysis of the relationship between colorectal serrated lesions and serum lipid levels. It provided reliable evidence and a scientific basis for identifying high risk populations with colorectal serrated lesions and the early clinical prevention and treatment of CRC.

## Methods

### Search strategy

We conducted the standard method according to the Preferred Reporting Items for Systematic Reviews and Meta-Analysis (PRISMA) guidelines. Two independent authors (XW and YZ) searched PubMed, Embase, Web of Science, the Cochrane Library, China Biomedical Literature Database, CNKI, Wanfang Database, and VIP Database for related studies comparing serum lipid levels in controls and patients suffering from colorectal serrated lesions. The keywords included lipoprotein, cholesterol, triglyceride, hyperlipidemia, dyslipidemia, intestinal polyp, adenomatous polyp, sessile serrated adenoma, sessile serrated polyp, and traditional serrated adenoma. Boolean logic operator “OR” was used to connect the search terms related to blood lipid level and the search terms related to serrated lesions, while “AND” was used to connect the search terms related to blood lipid level and serrated lesions. For example, the search strategy in the PubMed database was (“cholesterol” [Title/Abstract] OR “lipoprotein” [Title/Abstract] OR “triglyceride” [Title/Abstract] OR “hyperlipidemia” [Title/Abstract] OR “dyslipidemia” [Title/Abstract]) AND (“intestinal polyp” [Title/Abstract]) OR “adenomatous polyp” [Title/Abstract] OR “sessile serrated adenoma” [Title/Abstract] OR “sessile serrated polyp” [Title/Abstract] OR “traditional serrated adenoma” [Title/Abstract]). According to the different requirements of the different databases, the connectives were adjusted. In addition, manually retrieved references from the review articles and meta-analyses to supplement the literature were included in this study.

### Inclusion criteria and exclusion criteria

Inclusion criteria were the following: *1*) the subject was human; *2*) the patients were diagnosed with polyps or adenomas of the colon and/or rectum; and *3*) serum lipid levels included at least one of TC, TG, HDL-C, and low-density lipoprotein cholesterol (LDL-C).

Exclusion criteria were the following: *1*) the published literature had been repeated or had a potential duplicate publication; *2*) the article type was a review, conference abstract, editorial, note, or case report; *3*) the language was not Chinese or English; *4*) there was no case group or control group; *5*) the results did not contain blood lipid concentration or had missing sample size data; and *6*) the data for serrated lesions were not listed separately, or the data for non-serrated lesions could not be removed.

Two independent authors (XW and YZ) screened all the literature included in this study by title, abstract, and full text. The literature meeting the inclusion and exclusion criteria were finally determined. A third author (RZ) made the final decision for any discrepancy.

### Data extraction and literature quality evaluation

Data were extracted independently by the two authors (XW and YZ), and conflicts were resolved by the third author (RZ). The extracted data included the first author, year of publication, the state of the author, research type, colonoscopy time, type of serrated lesion, definition of the control group, a family history of CRC and colorectal polyps, history of inflammatory bowel disease, history of colorectal polyp, history of CRC, history of colorectal surgery, lipid-lowering drug-taking, the odds ratio (OR) of smoking and drinking, and the age and sex of the subjects. In addition, the mean, median, and standard deviation of serum lipid levels (TC, TG, HDL-C, and LDL-C) in the case and control groups were extracted. The mean variance estimation (hkbu.eu.hk) was used to transform the median data into mean and standard deviation.

Although some studies divided the subjects into a case group and control group, the temporal sequence of the colonoscopy and lipid level measurement was not clearly clarified. An article was classified as a cohort study when it investigated the incidence or prevalence of disease based on population characteristics or measurement parameters, while articles were classified as a case-control study when they grouped subjects before measuring the lipid levels. Otherwise, articles were classified as a cross-sectional study.

The Newcastle-Ottawa scale (NOS) was used to evaluate the quality of a case-control study or a cohort study. When the score was >7, the study was considered to be of high quality. The Agency for Healthcare Research and Quality (AHRQ) scale was used to evaluate the quality of the cross-sectional studies. When the score was >8, the study was considered to be of high quality.

### Statistical analysis

The extracted data were transformed. Lipid unit conversion (medsci.cn) was used to convert the lipid concentration units to mmol/L. The combined means and SDs (cuhk.edu.HK) were used to merge each group’s mean and standard deviation.

Review Manager 5.0 was used for statistical analysis. Weighted mean differences (WMDs) and 95% confidence intervals (CIs) were computed. *I*-squared (*I* ^2^) was used to assess the heterogeneity of the results. When *I* ^2^ **<** 5 0%, a fixed-effect model was used for the meta-analysis. When 50% ≤ *I* ^2^ < 75%, a random-effect model was used for the meta-analysis. When *I* ^2^ ≥ 75%, the source of the heterogeneity should be analyzed. Subgroup analyses and meta-regression analyses were performed to identify the potential sources of the heterogeneity. The moderators selected for this study included the subjects’ baseline characteristics and pathological types, risk factors for colorectal serrated lesions, factors influencing the diagnosis of colorectal serrated lesions, and factors influencing lipid levels. Sensitivity analysis was performed by one-by-one elimination to assess the stability of the results. The Egger test was used to evaluate the publication bias.

## Results

### Literature selection and exclusion

**FIGURE 1:**
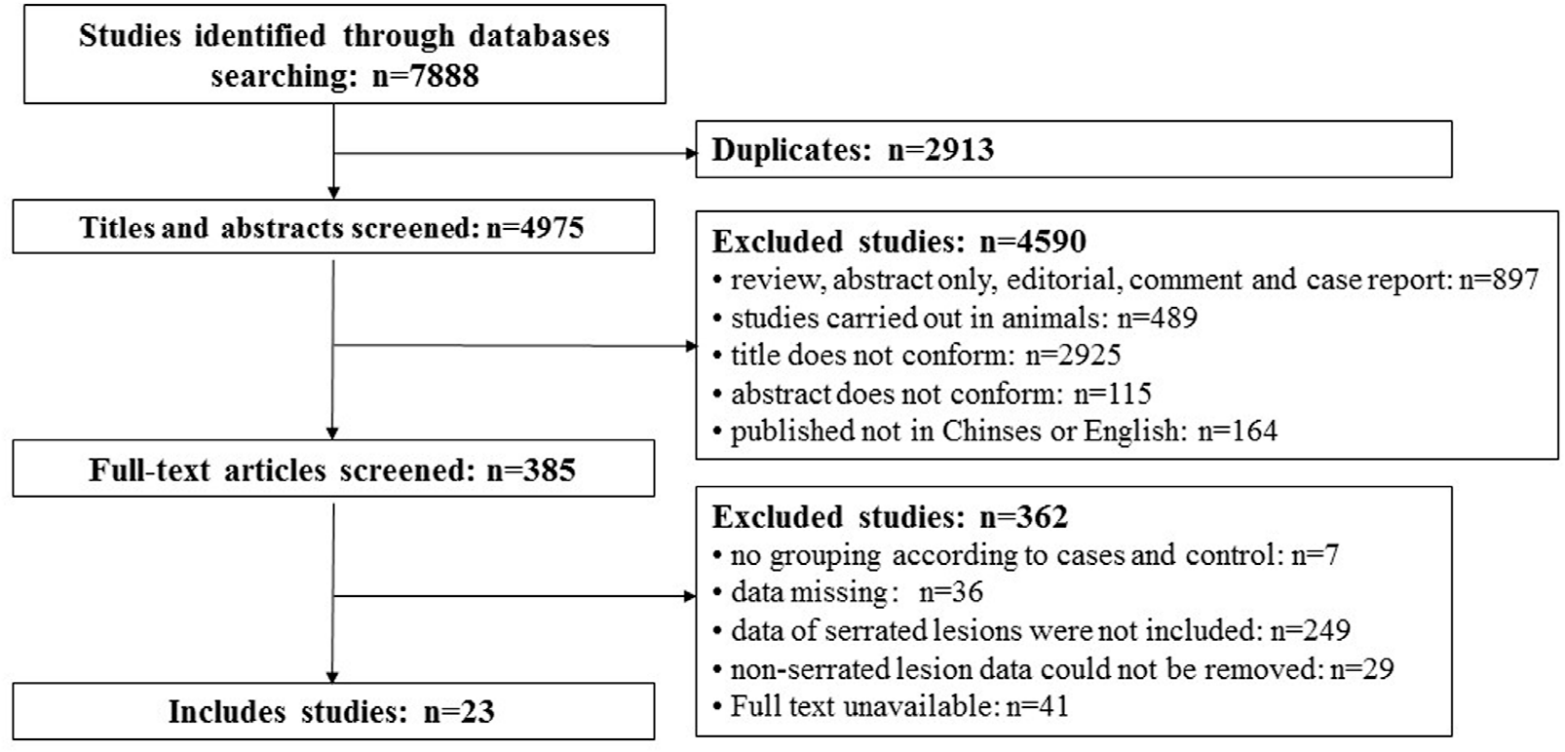
Flow diagram of literature include and exclude.

A total of 7,888 articles were searched out. There were 2,913 repeat studies. Then, 4,590 articles were excluded according to the title and abstract, and 362 articles were excluded after reading the full text. Finally, 23 articles were included. The flowchart of the literature selection and exclusion are shown in Figure 1.

### Characteristics of the included literatures

The characteristics of the included literature are shown in Table 1. A total of 65,972 subjects were included, including 2,063 cases with colorectal serrated lesions and 63,909 controls. Most of the subjects were from Asia, and most of the serrated lesions were hyperplastic polyps. The 23 studies included 19 case-control studies, one cohort study, and three cross-sectional studies.

**TABLE 1:** The characteristics of the included studies.

First author and year of publication	Country	Design	Year(s) of colonoscopy	Sample size (case/control)	Age (year)	Male (%)	Pathologic types	Definition of controls (Polyp-free)	Serum lipids	confounding factor for adjustment	Quality score
Ding et al. (2015)	China	Case-control study	2014–2014	31/726	48.04 ± 7.97	67.24	hyperplastic polyps	Normal	TC, TG, HDL-C, LDL-C	None	5
Zhu (2017)	China	Case-control study	2015–2016	40/318	50.80 ± 14.25^a^	40.88^a^	TSA	Normal	TG	None	5
Wang et al. (2005)	China	Cross-sectional study	2001–2002	138/4122	48.53 ± 11.47	54.3	hyperplastic polyps	Normal	TG, HDL-C	None	3
Liu et al. (2010)	China	Cross-sectional study	2006–2008	341/3062	48.53 ± 11.47	55.19	hyperplastic polyps	Normal	TC, TG, HDL-C	AGE	7
Fliss-Isakov et al. (2017)	Israel	Case-control study	2010–2015	75/407	57.71 ± 6.71	46.27	hyperplastic polyps, TSA	Normal	TG, HDL-C	AGE, SEX	7
Xie et al. (2019)	China	Cohort study	2015–2017	14/48	44.81 ± 11.17^a^	50^a^	hyperplastic polyps	Normal	TC, TG, HDL-C, LDL-C	None	6
Pyo et al. (2018)	Korea	Case-control study	2002–2012	395/34730	48.55 ± 9.31	50.5^a^	SSA/P, TSA	Normal	TC, TG, HDL-C, LDL-C	SEX	5
Anderson et al. (2011)	USA	Case-control study	2007–2010	90/200	N	39.66	SSA/P	Normal	TC, TG	None	5
Kim et al. (2019)	Korea	Cross-sectional study	2012–2017	423/18277	47.03 ± 10.61	40.86	SSA/P, TSA	Normal	TC, TG, HDL-C, LDL-C	SEX	5
Wen and Wang (2018)	China	Case-control study	2016–2017	32/50	46.7^a^	54^a^	hyperplastic polyps	Normal	TC, TG, HDL-C, LDL-C	AGE, SEX	7
Zheng et al. (2018)	China	Case-control study	2016–2018	5/60	54.37 ± 12.96^a^	38.33^a^	hyperplastic polyps	Normal	TC, TG, HDL-C, LDL-C	None	5
Lin et al. (2014)	China	Case-control study	2011–2012	33/138	49.3 ± 12.4^a^	61.59^a^	hyperplastic polyps	No polyps	TC, TG, LDL-C	AGE, SEX	7
Cao et al. (2015)	China	Case-control study	2012–2015	59/153	62.3^a^	54.9^a^	hyperplastic polyps	Normal	TC, TG, LDL-C	AGE, SEX	7
Ying et al. (2019)	China	Case-control study	2014–2015	81/628	45.34 ± 10.05	61.92	hyperplastic polyps	Normal	TC, TG, LDL-C	None	5
Zhang (2015)	China	Case-control study	2011–2014	25/120	50 ± 12^a^	63.33^a^	hyperplastic polyps	No polyps	TC, TG, LDL-C	AGE, SEX	7
Zhou et al. (2015)	China	Case-control study	2012–2014	30/112	57 ± 14^a^	57.14^a^	hyperplastic polyps	No polyps	TC, TG, HDL-C, LDL-C	AGE, SEX	7
Pan (2019)	China	Case-control study	2018–2018	11/104	54.06 ± 19.57^a^	59.62^a^	hyperplastic polyps	Normal	TC, TG, HDL-C, LDL-C	AGE, SEX	7
Yang (2021)	China	Case-control study	2019–2020	19/82	60.42 ± 3.43^a^	62.2^a^	hyperplastic polyps	Normal	TC, TG, HDL-C, LDL-C	None	5
Dai et al. (2017)	China	Case-control study	2012–2015	100/103	46.55 ± 12.79	61.58	hyperplastic polyps, SSA/P, TSA	Normal	TC, TG, HDL-C, LDL-C	None	5
Ning (2018)	China	Case-control study	2016–2017	26/177	58.31 ± 12.22^a^	59.32^a^	hyperplastic polyps	Normal	TC, TG, HDL-C, LDL-C	AGE, SEX	7
Wu et al. (2021)	China	Case-control study	2019–2020	34/80	46.8 ± 13.4^a^	43.75^a^	hyperplastic polyps	Normal	TC, TG, HDL-C, LDL-C	None	5
Zhang and Li (2014)	China	Case-control study	2013–2014	45/164	46.7 ± 9.8^a^	62.8^a^	hyperplastic polyps	No polyps	TC, TG, HDL-C, LDL-C	AGE, SEX	7
Gong (2018)	China	Case-control study	2017–2018	16/48	44.81 ± 11.17^a^	50^a^	hyperplastic polyps	Normal	TC, TG, HDL-C, LDL-C	None	5

a Data related to controls only.

**FIGURE 2:**
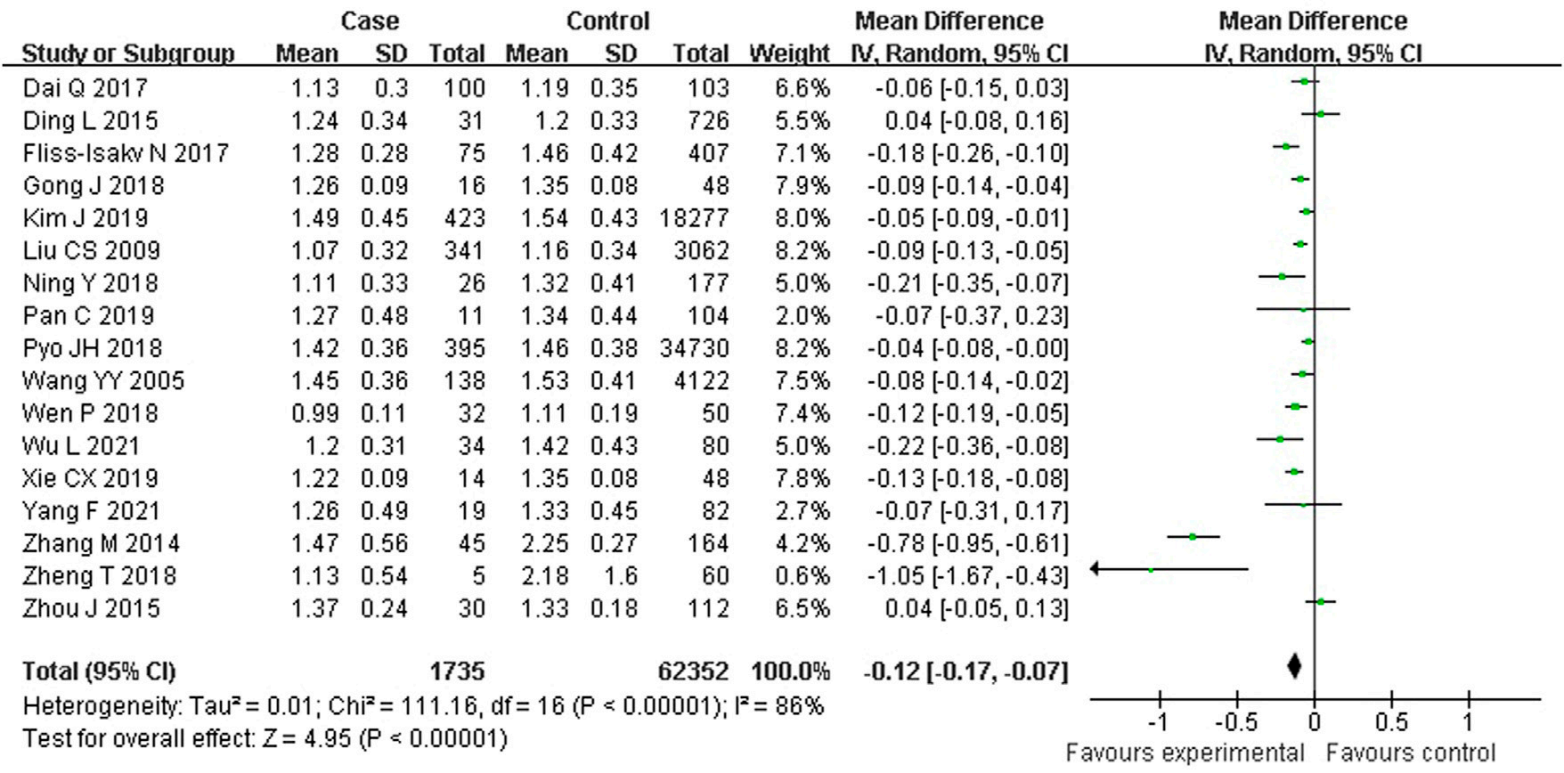
Forest plot of meta-analysis for association of serum HDL-C and colorectal serrated lesions.

As shown in Figure 2, 17 studies reported an association between serum HDL-C and colorectal serrated lesions in a population of 1,735 patients and 62,352 controls. The meta-analysis showed that the serum HDL-C level was lower in patients with colorectal serrated lesions than in the controls (WMD = −0.122 mmol/L, 95% CI = −0.170–0.073, *p* < 0.001). Because the heterogeneity test result showed *I* ^*2*^ = 85.6% (*p* < 0.001), suggesting high heterogeneity, a meta-regression and subgroup analysis were performed to explore the source of the heterogeneity. The results suggested that the undefined healthy subjects in the control group were the main source of the heterogeneity. Fifteen studies showed lower HDL-C levels in the case group, suggesting that the population with colorectal serrated lesions may have had lower HDL-C levels (Table 2).

**TABLE 2:** Heterogeneity source analyses for mean difference of HDL-C.

Variables	NO. of studies	*WMD*(95%*CI*) (mmol/L)	Heterogeneity		Meta-regression *p*
			*I* ^2^ *(%)*	*p*	
Chinese					
Yes	14	−0.138 (−0.201, −0.075)	86.1	<0.001	0.623
No	3	−0.082 (−0.148, −0.015)	82.0	0.004	
Case-control study					
Yes	13	−0.150 (−0.228, −0.073)	88.6	<0.001	0.540
No	4	−0.086 (−0.118, −0.054)	45.0	0.142	
Hyperplastic polyps					
Yes	14	−0.148 (−0.211, −0.085)	86.5	<0.001	0.393
No	3	−0.045 (−0.072, −0.019)	0.0	0.890	
Normal control					
Yes	15	−0.096 (−0.127, −0.064)	64.0	<0.001	0.166
No	2	−0.367 (−1.170, 0.437)	98.6	<0.001	
Excluded subjects with family history of CRC					
Yes	4	−0.071 (−0.113, −0.028)	61.5	0.050	0.612
No	13	−0.143 (−0.213, −0.074)	87.3	<0.001	
Excluded subjects with family history of CRA					
Yes	3	−0.103 (−0.170, −0.036)	45.5	0.160	0.840
No	14	−0.127 (−0.184, −0.069)	87.9	<0.001	
Excluded subjects with history of inflammatory bowel					
Yes	12	−0.142 (−0.210, −0.075)	89.1	<0.001	0.527
No	5	−0.091 (−0.144, −0.037)	59.0	0.045	
Excluded subjects with history of CRA					
Yes	7	−0.109 (−0.156, −0.062)	71.8	0.002	0.865
No	10	−0.130 (−0.218, −0.042)	90.0	<0.001	
Excluded subjects with history of CRC					
Yes	16	−0.131 (−0.184, −0.078)	85.5	<0.001	0.613
No	1	−0.040 (−0.076, −0.004)	—	—	
Excluded subjects with history of colorectal surgery					
Yes	3	−0.083 (−0.125, −0.040)	0.0	0.843	0.558
No	14	−0.135 (−0.192, −0.077)	88.3	<0.001	
Excluded subjects taking lipid-lowering drugs					
Yes	10	−0.176 (0.277, −0.076)	90.2	<0.001	0.364
No	7	−0.083 (−0.118, −0.048)	60.1	0.020	
The OR of smoking >1					
Yes	4	−0.076 (−0.130, −0.023)	73.1	0.011	0.504
No	13	−0.146 (−0.214, −0.079)	87.1	<0.001	
The OR of drinking >1					
Yes	3	−0.045 (−0.072, −0.019)	0.0	0.890	0.393
No	14	−0.148 (−0.211, −0.085)	86.5	<0.001	
Comparable in age					
Yes	7	−0.079 (−0.118, −0.039)	92.2	<0.001	0.397
No	10	−0.192 (−0.309, −0.075)	65.9	0.002	
Comparable in sex					
Yes	8	−0.160 (−0.255, −0.065)	92.3	<0.001	0.647
No	9	−0.094 (−0.135, −0.052)	58.9	0.013	

**FIGURE 3:**
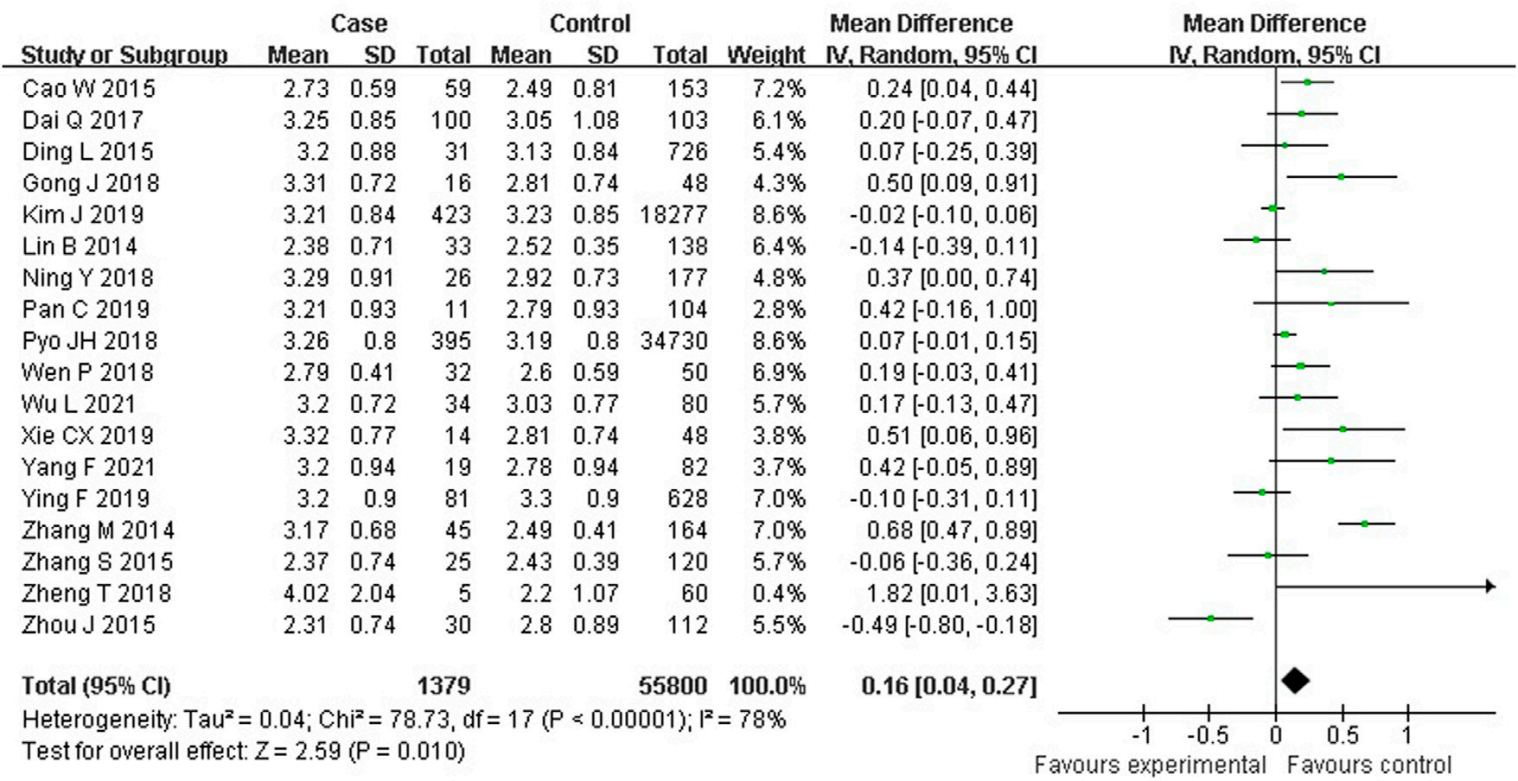
Forest plot of meta-analysis for association of serum LDL-C and colorectal serrated lesions.

As shown in Figure 3, 18 studies reported an association between serum LDL-C and colorectal serrated lesions in a population of 1,379 patients and 55,800 controls. The meta-analysis showed that the serum LDL-C level was higher in patients with colorectal serrated lesions than in the controls (WMD = 0.155 mmol/L, 95% CI = 0.038–0.273, *p* = 0.010). The result of the heterogeneity test showed *I* ^*2*^ = 78.4% (*p* < 0.001), suggesting high heterogeneity. The results of a meta-regression and subgroup analysis suggested that the undefined healthy subjects in the control group and the unexcluded subjects with a family history of CRC were the main source of the heterogeneity. Thirteen studies showed higher LDL-C levels in the case group, suggesting that the population with colorectal serrated lesions may have had higher LDL-C levels (Table 3).

**TABLE 3:** Heterogeneity source analyses for mean difference of LDL-C.

Variables	NO. of studies	*WMD*(95%*CI*) (mmol/L)	Heterogeneity		Meta-regression *p*
			*I* ^2^ *(%)*	*p*	
Chinese					
Yes	16	0.193 (0.030, 0.355)	77.5	<0.001	0.438
No	2	0.025 (−0.063, 0.114)	58.7	0.120	
Case-control study					
Yes	16	0.160 (0.023, 0.297)	77.7	<0.001	0.972
No	2	0.196 (−0.315, 0.706)	80.3	0.024	
Hyperplastic polyps					
Yes	15	0.194 (0.019, 0.369)	79.0	<0.001	0.543
No	3	0.042 (−0.047, 0.132)	49.8	0.136	
Normal control					
Yes	14	0.158 (0.062, 0.255)	56.9	0.004	0.288
No	4	0.005 (−0.520, 0.529)	93.8	<0.001	
Excluded subjects with family history of CRC					
Yes	4	0.077 (−0.042, 0.197)	63.6	0.041	0.878
No	14	0.176 (−0.004, 0.356)	79.7	<0.001	
Excluded subjects with family history of CRA					
Yes	3	0.246 (0.067, 0.424)	0.0	0.398	0.521
No	15	0.134 (0.003, 0.265)	80.9	<0.001	
Excluded subjects with history of inflammatory bowel					
Yes	13	0.126 (−0.040, 0.291)	83.0	<0.001	0.394
No	5	0.209 (0.043, 0.376)	44.9	0.123	
Excluded subjects with history of CRA					
Yes	6	0.168 (0.001, 0.335)	71.0	0.004	0.942
No	12	0.132 (−0.060, 0.323)	80.9	<0.001	
Excluded subjects with history of CRC					
Yes	17	0.171 (0.028, 0.313)	79.6	<0.001	0.724
No	1	0.070 (-0.009, 0.149)	_	_	
Excluded subjects with history of colorectal surgery					
Yes	3	0.313 (0.112, 0.515)	0.0	0.430	0.289
No	15	0.123 (−0.004, 0.251)	80.5	<0.001	
Excluded subjects taking lipid-lowering drugs					
Yes	12	0.215 (−0.004, 0.433)	81.2	<0.001	0.512
No	6	0.059 (−0.033, 0.152)	50.7	0.072	
The OR of smoking >1					
Yes	4	0.024 (-0.061, 0.109)	45.0	0.141	0.277
No	14	0.221 (0.037, 0.405)	78.0	<0.001	
The OR of drinking >1					
Yes	4	0.024 (−0.061, 0.109)	45.0	0.141	0.277
No	14	0.221 (0.037, 0.405)	78.0	<0.001	
Comparable in age					
Yes	8	0.146 (−0.113, 0.404)	86.2	<0.001	0.813
No	10	0.117 (0.007, 0.228)	58.3	0.010	
Comparable in sex					
Yes	10	0.114 (−0.038, 0.267)	85.4	<0.001	0.417
No	8	0.224 (0.033, 0.415)	55.4	0.028	

**FIGURE 4:**
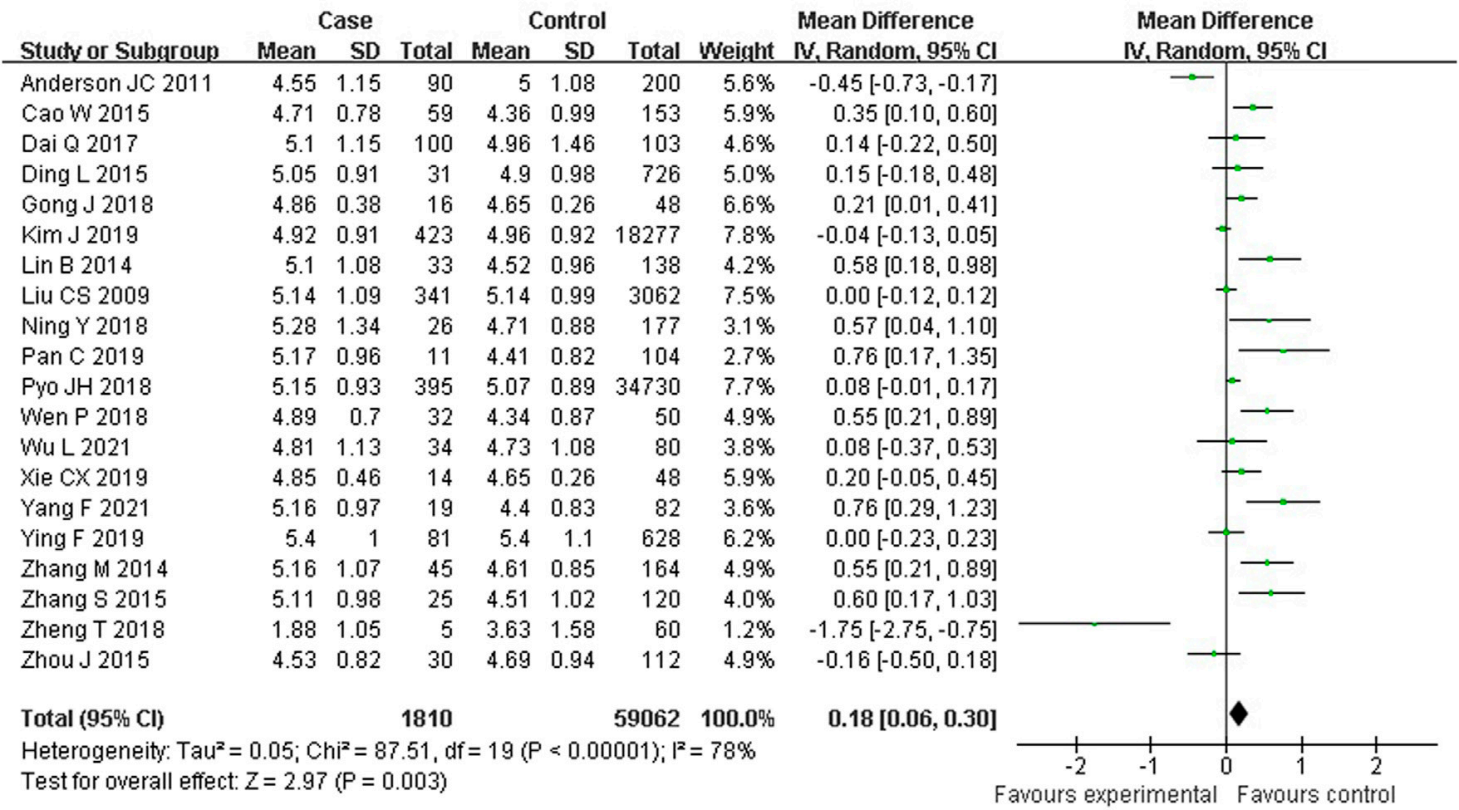
Forest plot of meta-analysis for association of serum TC and colorectal serrated lesions.

As shown in Figure 4, 20 studies reported an association between serum TC and colorectal serrated lesions in a population of 1,810 patients and 59,062 controls. The meta-analysis showed that the serum TC level was higher in patients with colorectal serrated lesions than in controls (WMD = 0.180 mmol/L, 95% CI = 0.061–0.299, *p* < 0.001). The result of the heterogeneity test showed *I* ^*2*^ = 78.3% (*p* < 0.001), suggesting high heterogeneity. The meta-regression and subgroup analysis suggested that the unexcluded subjects taking lipid-lowering drugs and other drugs that affect blood lipid levels were the main source of the heterogeneity. Sixteen studies showed higher TC levels in the case group, suggesting that the population with colorectal serrated lesions may have had higher TC levels (Table 4).

**TABLE 4:** Heterogeneity source analyses for mean difference of TC.

Variables	NO. of studies	*WMD*(95%*CI*) (mmol/L)	Heterogeneity		Meta-regression *p*
			*I* ^2^ *(%)*	*p*	
Chinese					
Yes	17	0.259 (0.111, 0.406)	72.9	<0.001	0.095
No	3	−0.083 (−0.270, 0.104)	85.2	0.001	
Case-control study					
Yes	17	0.221 (0.063, 0.380)	78.2	<0.001	0.477
No	3	0.004 (−0.091, 0.100)	36.4	0.208	
Hyperplastic polyps					
Yes	16	0.266 (0.110, 0.423)	74.6	<0.001	0.111
No	4	−0.049 (0.216, 0.117)	78.8	0.003	
Normal control					
Yes	16	0.134 (0.012, 0.255)	77.2	<0.001	0.321
No	4	0.383 (0.001, 0.765)	76.0	0.006	
Excluded subjects with family history of CRC					
Yes	5	−0.008 (−0.161, 0.145)	76.2	<0.001	0.137
No	15	0.274 (0.103, 0.446)	77.4	0.001	
Excluded subjects with family history of CRA					
Yes	3	0.178 (0.015, 0.342)	0.0	0.850	0.865
No	17	0.187 (0.052, 0.322)	81.3	<0.001	
Excluded subjects with history of inflammatory bowel					
Yes	14	0.161 (0.022, 0.301)	75.1	<0.001	0.644
No	6	0.259 (−0.036, 0.553)	85.5	<0.001	
Excluded subjects with history of CRA					
Yes	7	0.104 (−0.084, 0.292)	80.8	<0.001	0.401
No	13	0.237 (0.067, 0.408)	77.0	<0.001	
Excluded subjects with history of CRC					
Yes	18	0.232 (0.097, 0.367)	76.9	<0.001	0.157
No	2	−0.168 (−0.686, 0.351)	91.9	<0.001	
Excluded subjects with history of colorectal surgery					
Yes	3	0.319 (0.016, 0.622)	59.8	0.083	0.513
No	17	0.155 (0.027, 0.284)	79.3	<0.001	
Excluded subjects taking lipid-lowering drugs					
Yes	12	0.268 (0.082, 0.454)	68.6	<0.001	0.364
No	8	0.065 (−0.074, 0.203)	79.1	<0.001	
The OR of smoking >1					
Yes	5	−0.038 (−0.176, 0.101)	71.7	0.007	0.070
No	15	0.290 (0.122, 0.457)	75.3	<0.001	
The OR of drinking >1					
Yes	4	0.022 (−0.055, 0.098)	22.6	0.275	0.378
No	16	0.236 (0.058, 0.413)	80.2	<0.001	
Comparable in age					
Yes	9	0.384 (0.157, 0.611)	78.0	<0.001	0.060
No	11	0.048 (−0.091, 0.187)	76.1	<0.001	
Comparable in sex					
Yes	10	0.316 (0.143, 0.489)	81.2	<0.001	0.105
No	10	0.045 (−0.140, 0.229)	76.6	<0.001	

**FIGURE 5:**
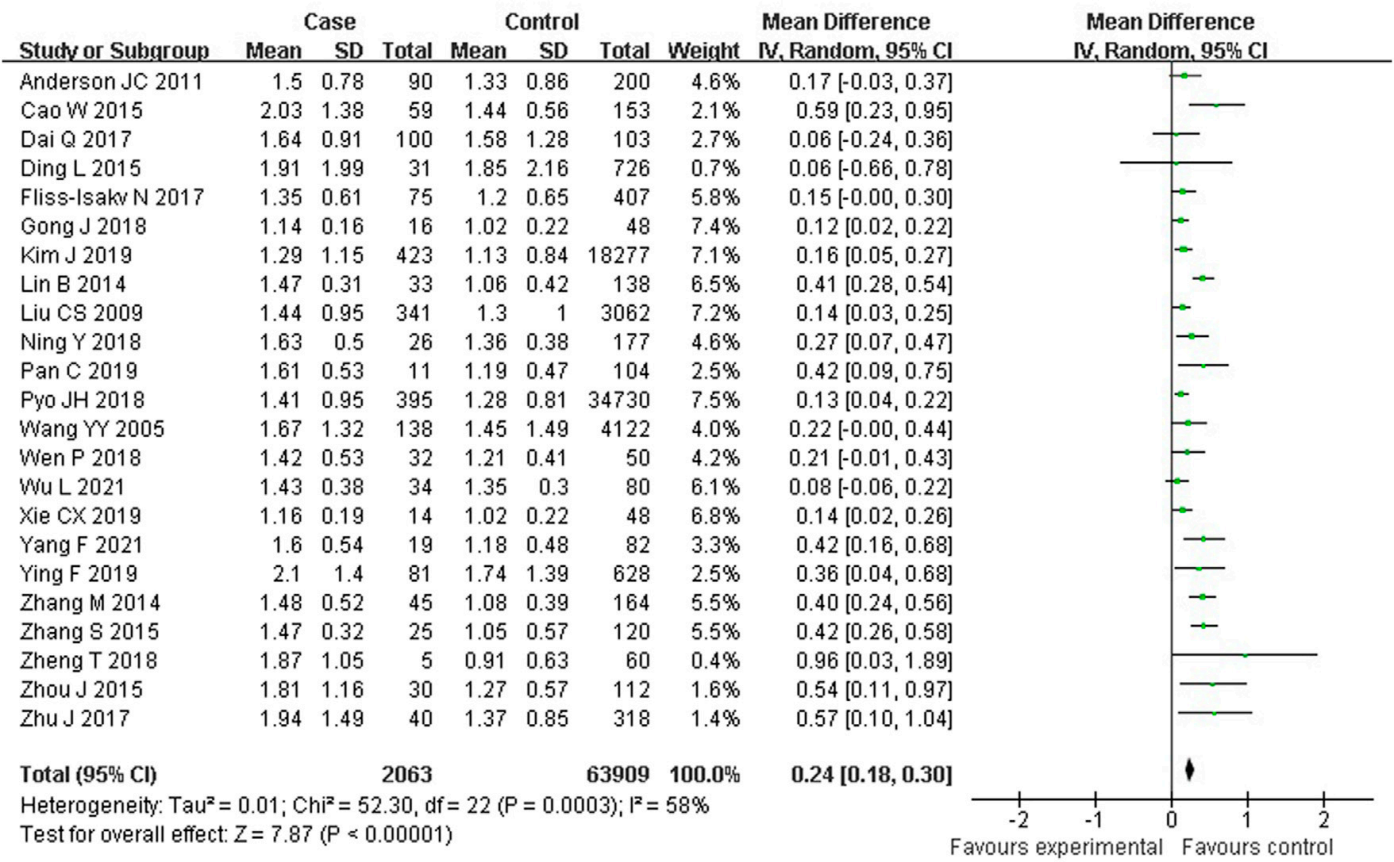
Forest plot of meta-analysis for association of serum TG and colorectal serrated lesions.

As shown in Figure 5, 23 studies reported an association between serum TG and colorectal serrated lesions in a population of 2,063 patients and 63,909 controls. Since the result of the heterogeneity test showed *I* ^*2*^ = 57.9% (*p* < 0.001), a random effect model was used for the meta-analysis. The meta-analysis showed that the serum TG level was higher in patients with colorectal serrated lesions than in the controls (WMD = 0.241 mmol/L, 95% CI = 0.181–0.302, *p* < 0.001).

### Sensitivity analysis and publication bias

The sensitivity analysis results showed good stability of the meta-analysis results on the relationship between the four blood lipid indexes (TC, TG, HDL-C and LDL-C) and colorectal serrated lesions (Table 5). The Egger test showed that there was no obvious publication bias in the literature included in the meta-analysis on the relationship between TC, HDL-C, LDL-C (*p* = 0.108) and colorectal serrated lesions. However, there might be a publication bias in the literature included in the meta-analysis of TG and colorectal serrated lesions (Table 6).

**TABLE 5:** The pooled results of sensitivity analyses [WMD (95% CI)].

Indicators	Minimum estimate (mmol/L)	Maximum estimate (mmol/L)	Overall result (mmol/L)
TC	0.1571 (0.0403, 0.2739)	0.2135 (0.0982, 0.3288)	0.1799 (0.0612, 0.2985)
TG	0.2243 (0.1670, 0.2815)	0.2519 (0.1884, 0.3153)	0.2414 (0.1813, 0.3016)
HDL-C	−0.1324 (−0.1815, −0.0832)	−0.0883 (−0.1207, −0.0558)	−0.1216 (−0.1697, −0.0735)
LDL-C	0.1010 (0.0027, 0.1994)	0.1884 (0.0751, 0.3017)	0.1552 (0.0376, 0.2727)

**TABLE 6:** The results of Egger test.

Indicators	Bias coefficient	*p*
TC	1.4306	0.086
TG	1.7309	0.009
HDL-C	−2.1838	0.072
LDL-C	1.3892	0.108

## Discussion

CRC, whose morbidity and mortality are high, can be a serious threat to human health. Colorectal adenomas are generally considered to be precancerous lesions of CRC. The early detection, diagnosis, and treatment of colorectal serrated lesions can effectively prevent CRC occurrence. However, colorectal serrated lesions are difficult to detect by fecal occult blood tests and CT colonic imaging because they do not easily bleed, are often sessile, and are flatter than traditional adenomas. In recent years, colorectal serrated lesions have gradually become an emerging research focus, and more attention has been paid to these lesions. Although colonoscopy can intuitively examine intestinal lesions, the fuzzy boundary and flat shape of serrated lesions increase the difficulty and false-negative rate by microscopic detection. Therefore, identifying populations at high risk for colorectal serrated lesions is of great significance.

Obesity, dietary fat, and total energy intake, which are closely related to hyperlipemia, have been proven to be associated with an increased risk of colorectal serrated lesions. Moreover, at present, many scholars regard dyslipidemia as one of the potential risk factors for colorectal cancer. Macarie M et al. found increased triglycerides levels were associated with the risk of sessile serrated lesions, which is important in the pathogenesis of colorectal carcinoma (Macarie et al., 2020). The relationship also had been proved in Japanese men (Tabuchi et al., 2006). The mechanisms include the following: *1*) the activation of insulin-like growth factor 1, which can be affected by TG, can inhibit apoptosis and promote the occurrence of tumors (Yang et al., 2013); *2*) dyslipidemia can induce the production of inflammatory cytokines (such as IL-6 and TNF-α) and reduce the secretion of anti-inflammatory cytokines (such as IL-10), thus creating a tumor-friendly cell environment and promoting cell proliferation (Esteve et al., 2005; Kim et al., 2017); *3*) chronic inflammation can further affect the normal cholesterol transport and stimulate compensatory changes, such as the synthesis LDL-C and very low-density lipoprotein cholesterol (VLDL-C), resulting in the accumulation of TG in intestinal cells (Esteve et al., 2005); *4*) a high fat diet can stimulate the secretion of bile acids, and secondary bile acids can further stimulate the proliferation of colorectal epithelial cells and inhibit the detoxification of exogenous carcinogens (Li et al., 2011); *5*) serum lipids can induce the oxidative stress of tissue cells, resulting in an increased production of reactive oxygen species and the abnormal expression of cancer-related genes (Xie et al., 2019); and *6*) cyclooxygenase-2, which can be activated by fatty acids and triglycerides, is related to the occurrence of CRC (Sasai et al., 2000).

Colorectal serrated lesions are the precancerous lesions of CRC. At present, there is no uniform conclusion on the relationship between serum lipid levels and colorectal serrated lesions. The present study conducted a systematic review and meta-analysis, including 65,972 subjects in 23 studies, to explore the evidence-based relationship between colorectal serrated lesions and blood lipid levels. The results showed that the serum levels of TC and LDL-C in patients with colorectal serrated lesions were higher than those in healthy subjects, while the level of HDL-C was lower than in healthy subjects. However, there was significant heterogeneity in the results. Therefore, we conducted a subgroup analysis to clarify the source of the heterogeneity. The results of the subgroup analysis showed that people in the control group with chronic nonspecific colitis might have led to the increased heterogeneity in the WMD of HDL-C, while people in the control group with chronic nonspecific colitis and with a family history of CRC might have been the main source of heterogeneity in the WMD of LDL-C. It was suggested that the serum levels of HDL-C and LDL-C in patients with chronic nonspecific colitis may be different from those in healthy people. In addition, a family history of CRC, which may be another risk factor for colorectal serrated disease, might have led to the bias of the present study’s results. In addition, lipid-lowering drugs may affect serum TC levels. Excluding the subjects taking lipid-lowering drugs could reduce the heterogeneity of the WMD of TC. Among the included studies, the results of most studies showed that the serum levels of TC and LDL-C in patients with colorectal polyps were higher than in healthy people. However, the HDL-C level was lower than in healthy people. This was consistent with the results of the meta-analysis, further proving the reliability of the results.

There were some limitations of this study. First, the results of the Egger test showed possible publication bias in the literature included in the meta-analysis of TG and colorectal serrated lesions, indicating low credibility of the results. Second, most of the included studies were case-control studies with lower reliability than the cohort studies and randomized controlled trials. Third, studies on serrated lesions were limited, and some information could not be extracted from many of the included studies. This affected the credibility of the meta-regression analysis and subgroup analysis. Fourth, in some literature, the mean and standard deviation of the blood lipid levels were obtained by median transformation, while in other literature, the blood lipid levels and other extracted data were obtained by inter-group combination. This, too, maybe a partial source of heterogeneity. Fifth, the language of included study was Chinese or English, which may lead to most of studies from Asian. There might be a report bias exist. The above limitations may have impacted the results of the meta-analysis.

However, this study suggested a certain correlation between colorectal serrated lesions and serum lipid levels. Including people with dyslipidemia in the focus screening population for colorectal serrated lesions may improve the screening efficiency. In addition, paying more attention to the lipid levels of young people could also contribute to screening and preventing early-onset CRC. Moreover, clinicians should be more cautious when performing colonoscopies and diagnosing patients with hyperlipidemia. Besides, because many factors influence the occurrence of colorectal serrated lesions and blood lipid levels, and these factors may interact with each other, it is necessary to classify patients according to the pathological type of colorectal serrated lesions. It is also necessary to explore the differences in blood lipid levels of patients with different types of serrated lesions. Because only two articles in this study grouped patients into SSA and SSP, a meta-analysis comparing the different pathological types was not conducted in this study. However, a clearer classification is needed in clinical diagnosis in order to carry out a more in-depth study of each pathological type.

In conclusion, colorectal serrated lesions may be related to blood lipid levels. Hyperlipidemia might be a risk factor for colorectal serrated lesions.

## Data Availability

The original contributions presented in the study are included in the article/Supplementary Material, further inquiries can be directed to the corresponding authors.
